# Attenuated T Cell Responses to a High-Potency Ligand In Vivo

**DOI:** 10.1371/journal.pbio.1000481

**Published:** 2010-09-14

**Authors:** Emily Corse, Rachel A. Gottschalk, Michelle Krogsgaard, James P. Allison

**Affiliations:** 1 Department of Immunology, Howard Hughes Medical Institute and Ludwig Center for Cancer Immunotherapy, Memorial Sloan-Kettering Cancer Center, New York, New York, United States of America; 2 Immunology and Microbial Pathogenesis Program, Weill Cornell Graduate School of Medical Sciences, New York, New York, United States of America; 3 Department of Pathology and New York University (NYU) Cancer Institute, NYU School of Medicine, New York, New York, United States of America; National Jewish Medical and Research Center/Howard Hughes Medical Institute, United States of America

## Abstract

αβ T cell receptor (TCR) recognition of foreign peptides bound to major histocompatibility complex (pMHC) molecules on the surface of antigen presenting cells is a key event in the initiation of adaptive cellular immunity. In vitro, high-affinity binding and/or long-lived interactions between TCRs and pMHC correlate with high-potency T cell activation. However, less is known about the influence of TCR/pMHC interaction parameters on T cell responses in vivo. We studied the influence of TCR/pMHC binding characteristics on in vivo T cell immunity by tracking CD4^+^ T cell activation, effector, and memory responses to immunization with peptides exhibiting a range of TCR/pMHC half-lives and in vitro T cell activation potencies. Contrary to predictions from in vitro studies, we found that optimal in vivo T cell responses occur to ligands with intermediate TCR/pMHC half-lives. The diminished in vivo responses we observed to the ligand exhibiting the longest TCR/pMHC half-life were associated with attenuation of intracellular signaling, expansion, and function over a broad range of time points. Our results reveal a level of control over T cell activation in vivo not recapitulated in in vitro assays and highlight the importance of considering in vivo efficacy of TCR ligands as part of vaccine design.

## Abstract 2

According to this study, the strongest T cell receptor ligands in vitro do not necessarily induce the strongest T cell responses in vivo, suggesting that vaccine designers may need to reconsider their strategies.

## Author Summary

As an important part of immune system, T cells fight infections by recognizing signs of foreign invaders. A molecule on the surface of these cells—called the T cell receptor—recognizes and binds to protein components (peptides) from bacteria, viruses, and other pathogens that are displayed on the surface of other cells. The T cells can then use this information to orchestrate the fight against infection. Vaccination involves injecting into the body foreign peptides that mimic a pathogen, therefore tricking it into raising a T cell response against that pathogen that will be protective in the event of a real infection. We studied T cell responses in mice injected with one of several peptides to which the T cell receptor binds more or less strongly. Contrary to expectations, we found that the peptide that interacted most strongly with the T cell receptor did not provoke the strongest T cell response. This may be nature's way of preventing harmful inflammatory damage due to excessively strong T cell activation. Our work shows that peptides that bind the T cell receptor with medium strength may be best to use for vaccines. Current vaccine strategies seeking to design peptides that bind with maximum strength to the T cell receptor may need to be reconsidered in light of our findings.

## Introduction

Activation of T cells by peptides bound to major histocompatibility complex (pMHC) molecules on antigen presenting cells (APCs) initiates the development of adaptive immunity to pathogens and is characterized by relatively low affinity but highly sensitive interactions between TCRs and pMHC [1],[2]. Our understanding of the biochemical requirements for T cell activation by pMHC ligands has been advanced by the use of peptides that vary from the natural ligands at TCR contact residues [3],[4]. Since the initial report of a kinetic basis for TCR ligand discrimination [5], the affinity and half-life of TCR/pMHC interactions have been studied as important determinants of T cell activation potency. It has been generally accepted that pMHC ligands with greater affinity/slower off-rates result in a higher potency of T cell activation [6]–[9], although there are exceptions [10]. Insight into apparent discrepancies among studies that found the affinity [8],[9],[11] versus the half-life [6],[10],[12] of the TCR/pMHC interaction to be the most influential determinant of T cell activation potency has been provided by a recent report in which affinity (K_D_) was found to be the more closely associated with potency when the association rate (k_a_) was large (>10^5^ M^−1^s^−1^), whereas half-life or dissociation rate (k_d_) correlated well with potency when k_on_ was small (10^3^ M^−1^s^−1^) [13]. In both cases (small or large k_a_), the strongest interactions, whether defined by kinetic or equilibrium parameters, yielded the most potent T cell responses.

In addition to its influence upon the magnitude of T cell responses, TCR ligand potency is also thought to influence the outcome of in vitro CD4^+^ helper T cell differentiation [14],[15], as well as T cell motility during early stages of in vivo T cell activation [16],[17]. It is critical to understand how biochemical parameters of TCR recognition influence the entire course of an in vivo CD4^+^ T cell response, especially given that the goal of many vaccine strategies is to elicit strong, persistent T cell responses to pathogens and tumors. The importance of CD4^+^ T cell help in vaccination protocols is underscored by the suboptimal responses elicited by minimal CD8^+^ T cell epitopes in many cases [18].

We examined the effect of TCR ligand potency upon in vivo CD4+ T cell responses using adoptively transferred 5C.C7 T cells activated by immunization with lipopolysaccharide (LPS) and moth cytochrome c (MCC) peptide, or related ligands of varying potency of in vitro T cell activation [19]–[23], including one peptide initially identified as an in vitro superagonist ligand for the cytochrome c-reactive 2B4 TCR [24]. This strategy resulted in fully functional differentiation of effector and memory T cells and allowed us to evaluate the influence of TCR ligand potency on multiple stages of the T cell response, which is not possible using in vitro methods, in a system with few variables that is relevant to CD4^+^ T cell vaccine design [18]. In contrast to predictions from the in vitro hierarchy of ligand potency and the commonly held assumption that more potent T cell responses result from longer-lived TCR/pMHC interactions during T cell recognition, we observed an optimal in vivo T cell response in the middle of the avidity spectrum.

A recent study of CD8^+^ T cell responses to *Listeria monocytogenes* expressing various OT-1 TCR ligands [25] showed a good correlation between ligand potency in vitro and the magnitude of the T cell response in vivo, i.e. the natural OT-1 TCR ligand SIINFEKL exhibited the most potent responses in vitro and in vivo. In our study of in vivo CD4^+^ T cell responses, we were able to examine a ligand that is stronger in vitro than the natural 5C.C7 TCR ligand MCC (88–103) [24], and the blunted responses we observed to this ligand in vivo were associated with attenuation of signaling, proliferation, and function at several points during the T cell response, rather than increased cell death, overactivation, or clonal exhaustion. These results point to an upper limit of in vivo TCR ligand potency, which could serve to protect against deleterious inflammatory effects during responses to strong TCR stimulation, and highlight the importance of considering the in vivo efficacy of TCR ligands as part of vaccine strategies that aim to promote high-avidity T cell recognition of tumors or pathogens [11].

## Results

### Biochemical Determinants of 5C.C7 TCR Ligand Potency

**Figure 1:**
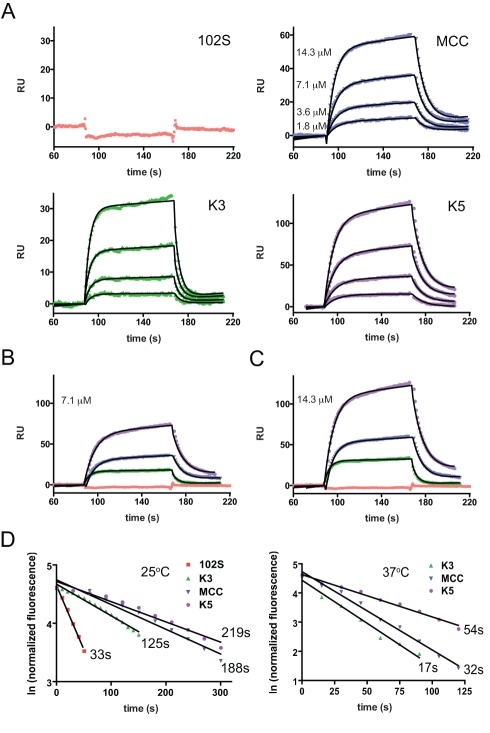
Kinetic parameters of the interaction of the 5C.C7 TCR with four peptide/I-E^k^ ligands. (A) Surface plasmon resonance at 25°C was used to study the interaction of varying concentrations of soluble 5C.C7 TCR with the indicated immobilized peptide/I-E^k^ complexes as described in Materials and Methods. The concentration of soluble TCR resulting in each RU trace is indicated on the MCC/I-E^k^ plot, and applies to the corresponding traces on the K3/I-E^k^ and K5/I-E^k^ plots. (B,C) Comparison of all four peptide/I-E^k^ complexes is shown at 7.1 µM and 14.3 µM TCR, respectively. Association (k_a_) and dissociation (k_d_) constants were calculated as described in Materials and Methods and are shown in Table 1. (D) Dissociation of PE-labeled H2-I-E^k^ tetramers bearing the indicated peptides from naïve 5C.C7 RAG2-deficient T cells was done at 25°C or 37°C. TCR/pMHC tetramer half-lives were calculated from linear regressions and are shown at the end of each line and in Table 1.

We used 5C.C7 TCR transgenic T cells, which recognize a peptide from MCC in the context of the mouse MHC class II molecule I-E^k^ [3], to study the effect of peptide/MHC ligand potency on in vivo CD4^+^ T cell activation. Amino acid substitutions of MCC (88–103) result in a panel of peptide ligands that bind to I-E^k^ equally well [24] but exhibit a range of potency for activation of 5C.C7 T cells in vitro [19]–[23]. Surface plasmon resonance (SPR) was used to study the kinetics of the interaction of purified 5C.C7 TCR with immobilized pMHC complexes (Figure 1). Figure 1A shows plots of resonance units (RU) after TCR injection for 102S/I-E^k^ (14.3 µM TCR), K3/I-E^k^ (1.8–14.3 µM TCR), MCC/I-E^k^ (1.8–14.3 µM TCR), and K5/I-E^k^ (1.8–14.3 µM TCR). SPR signal plots of all four pMHC complexes with 7.1 µM and 14.3 µM injected TCR are shown in Figure 1B and C, respectively. The observed SPR curves (shown in Figure 1 as colored symbols) were fitted to a 1∶1 Langmuir binding model (shown in Figure 1 as solid black lines) to calculate k_a_ and k_d_ constants, which were used to derive half-life and K_D_ of the TCR/pMHC interactions (Table 1). As shown in Figure 1A, the on- or off-rates of association of 5C.C7 with 102S/I-E^k^ were too fast to be accurately measured by SPR. The data indicate that MCC/I-E^k^ complexes have a slower off-rate than K3/I-E^k^ complexes, which corresponds to a half-life nearly twice as long (6.3 versus 3.4 s; see Table 1). K5/I-E^k^ complexes have the slowest off-rate, which results in a longer half-life than MCC/I-E^k^ complexes (9.1 versus 6.3 s; see Table 1). K_D_ values (resulting from k_d_/k_a_) for MCC/I-E^k^ and K5/I-E^k^ are similar (43.5 and 42.4 µM, respectively), while that for K3/I-E^k^ complexes (165 µM) indicates a lower monomeric binding affinity at 25°C (Table 1).

**Table 1:** In vitro properties of 5C.C7 TCR ligands.

Peptide Ligand	Sequence^a^	Ligand Affinity for I-E^k^ IC_50_ (nM)^b^	k_a_ (M^−1^s^−1^)^c^	k_d_ (s^−1^)^c^	K_D_ (µM)^c^	Monomer t_½_ (25°C) (s)^d^	Tetramer t_½_ (25°C) (s)^d^	Tetramer t_½_ (37°C) (s)^d^	In Vitro EC_50_ (nM)^e^
102S	ANERADLIAYLKQASK	113±4	nd^f^	nd^f^	nd^f^	nd^f^	33±0.4	nd^f^	160.3
K3	ANERADLIAYPKAATKF	90±2	1,230±236	0.203±0.00344	165	3.4	124.6±3.5	17.3±1.2	24.87
MCC	ANERADLIA*Y*L*K*QA*T*K	108±4	2,530±94.6	0.11±0.00134	43.5	6.3	188.1	31.9±1.3	37.80
K5	ANERADLIAYFKAATKF	100±3	1,810±83	0.0766±0.00108	42.4	9.1	219.4	53.6±8.3	17.52

a TCR contact residues are underlined and italicized in the MCC sequence.

b The binding affinity of soluble I-E^k^ for the various peptides was previously reported in [24]. The IC_50_ value is the concentration of unlabeled peptide that prevented 50% of biotinylated peptide binding to I-E^k^. Errors represent s.d. from the mean.

c Values were derived from SPR measurements obtained with immobilized peptide/I-E^k^ complexes and soluble 5C.C7 TCR. Data in Figure 1 were fitted to a 1∶1 Langmuir binding model to generate k_a_ and k_d_. K_D_ was calculated by k_d_/k_a_. Errors represent s.d. from the mean and are derived from two independent experiments.

d The half-life (t_1/2_) of I-E^k^ monomer binding to 5C.C7 TCR is equal to ln2/k_d_. k_d_ was calculated as described in note c. The half-life (t_1/2_) of I-E^k^ tetramer binding was calculated from linear regression plots (Figure 1) as the time at which median tetramer fluorescence was equal to 50% of that at *t* = 0. Errors represent s.d. from the mean, calculated from three or more independent experiments, except in the case of 102S 25°C (two independent experiments).

e EC_50_ values were determined as the peptide concentration that yielded 50% of maximal ^3^H-thymidine incorporation by 5C.C7 RAG2−/− lymphocytes in response to the indicated peptides presented by B10.A splenocytes (Figure 2A). Values are averaged from four independent experiments.

f Not determined. On- or off-rates were too fast to be measured accurately by SPR (see Figure 1). Thus, the half-life of 102S/I-E^k^ monomer binding to 5C.C7 TCR cannot be calculated. The half-life of 102S/I-E^k^ tetramer binding to 5C.C7 TCR was determined at 25°C but is too short to be measured at 37°C.

To examine the binding of the various pMHC ligands to 5C.C7 TCR in the context of live T cells, we measured the half-life of tetramer binding to naïve transgenic 5C.C7 T cells using PE-labeled I-E^k^ tetramers bearing the weak agonist peptide 102S [19],[20], the agonist peptides MCC and K3 [24], and the superagonist peptide K5 [22]–[24], as described in Materials and Methods (Figure 1D, Table 1). At 25°C (Figure 1D, left panel), 102S/I-E^k^ tetramers have the shortest half-life of TCR binding (33s), consistent with the inability to measure the interaction by SPR (Figure 1A) or by tetramer dissociation at 37°C (Figure 1D, right panel). K3/I-E^k^ tetramers have a shorter half-life of tetramer binding than MCC/I-E^k^ tetramers at both 25°C and 37°C. K5/I-E^k^ tetramers have the longest half-life of 5C.C7 TCR binding, consistent with the k_d_ and half-life derived by SPR (Table 1). The difference between K5/I-E^k^ and MCC/I-E^k^ tetramer dissociation is greatest at 37°C, with K5/I-E^k^ tetramers binding almost twice as long (Figure 1D, right panel). The half-lives of TCR/pMHC binding derived by tetramer dissociation are greater than those from SPR (Table 1), consistent with the interaction of multivalent versus monovalent pMHC complexes, but there is good agreement between the two methods when comparing relative off-rates of the various pMHC ligands (Figure S1), in accordance with previously published data [24],[26].

Although the SPR-derived K_D_ value for the binding of 5C.C7 TCR to monomeric K5/I-E^k^ complexes is only slightly lower than that for MCC/I-E^k^ (Table 1), the K5/I-E^k^ tetramer stains naïve 5C.C7 T cells more brightly at equilibrium (Figure S2A). As described in Materials and Methods, titrations of the tetramers at equilibrium were done as part of apparent K_D_ analysis (Figure S2B,C) [27], which shows that K5/I-E^k^ tetramers bind to 5C.C7 T cells with relatively higher avidity than MCC/I-E^k^ tetramers. The larger difference in the equilibrium K5 and MCC measurements seen with tetrameric pMHC and T cells (Figure S2) versus solution measurements with monomeric pMHC and TCR (Figure 1, Table 1) could be explained by a greater effective affinity due to the two-dimensional confinement imposed by the T cell membrane [28]–[30]. k_d_ is likely to be the more important determinant of pMHC ligand potency in this case [10], especially given the small association rates of the 5C.C7 TCR/pMHC complexes (k_a_ ∼10^3^ M^−1^s^−1^; Table 1) [13]. Taken together, the data indicate that the interaction of 5C.C7 TCR with K5/I-E^k^ complexes is stronger than with MCC-I-E^k^.

Consistent with previously published data showing that fewer K5/I-Ek complexes are needed to induce calcium flux in 5C.C7 T cells [22],[23], K5 peptide is a more potent inducer of T cell activation in vitro (Figure 2). Naïve 5C.C7 T cells were stimulated with splenocytes pulsed in vitro with the various peptides, and proliferation was assessed by incorporation of ^3^H-thymidine (Figure 2A). Approximately four times as much 102S peptide and half as much K5 peptide, on average, is needed to induce the same amount of T cell proliferation as the natural ligand MCC (in vitro EC_50_, Table 1), which reflects their previous characterization as weak agonist and superagonist, respectively [19]–[23]. K3 peptide seems to activate 5C.C7 T cells better than its half-life would suggest (Table 1), which could be analogous to results previously described for the related cytochrome c-reactive 2B4 TCR, in which K3 binding induced a heat capacity change that is potentially indicative of structural rearrangements that contribute to T cell activation potency [24]. We also activated naïve 5C.C7 T cells in vitro with DCs purified from the spleens of B10.A mice immunized with the various peptide ligands (Figure 2B–D), which shows that APCs pulsed with K5 peptide in vivo are the most potent stimulators of 5C.C7 T cells in vitro (see below).

**Figure 2:**
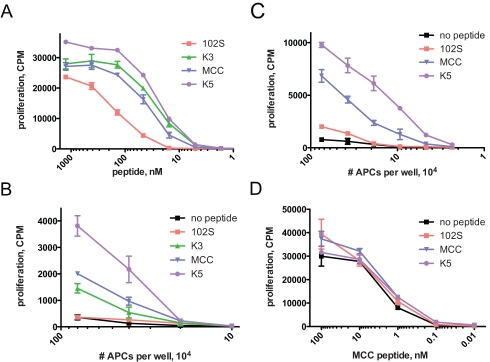
K5 peptide is a more potent inducer of in vitro 5C.C7 T cell proliferation than MCC peptide when presented on both in vitro and in vivo pulsed APCs. (A) Lymph node cells from 5C.C7 TCR transgenic RAG2−/− mice were stimulated with peptide-pulsed irradiated B10.A splenocytes in the presence of ^3^H-methyl-thymidine for 60 h. The mean EC_50_ for each peptide derived from these experiments is shown in Table 1. B10.A mice, three per peptide group, with (B) or without (C) adoptively transferred 5C.C7 RAG2−/− T cells, were immunized with the indicated peptides and LPS, and 2 d later CD11c+ splenocytes were purified, irradiated, and used to stimulate naive 5C.C7 RAG2−/− T cells in vitro for 60 h. (D) Titration of exogenous MCC peptide added to the in vivo–pulsed APCs in (C) (10^5^ per well) shows that the APCs are equally capable of presenting antigen. Data in Figure 2 are representative of at least three independent experiments. Error bars show mean ± s.e.m., *n* = 3 wells.

### Diminished In Vivo T Cell Responses to High-Avidity TCR Stimulation

**Figure 3:**
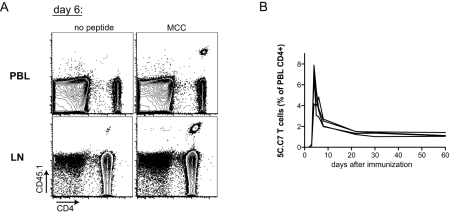
Immunization with MCC peptide and LPS results in expansion, contraction, and maintenance of 5C.C7 T cells in vivo. (A) B10.A mice with adoptively transferred naïve 5C.C7 RAG2−/− CD45.1 T cells were immunized with LPS alone (“no peptide”) or LPS+MCC peptide (“MCC”). CD4^+^CD45.1^+^ lymphocytes were detected in blood (“PBL”) and lymph node (“LN”) samples 6 d after immunization (LN CD4^+^ were purified by negative selection before flow cytometry). (B) Time course of 5C.C7 expansion, contraction, and maintenance in blood in response to immunization with MCC peptide. The traces of four individual mice are shown.

**Figure 4:**
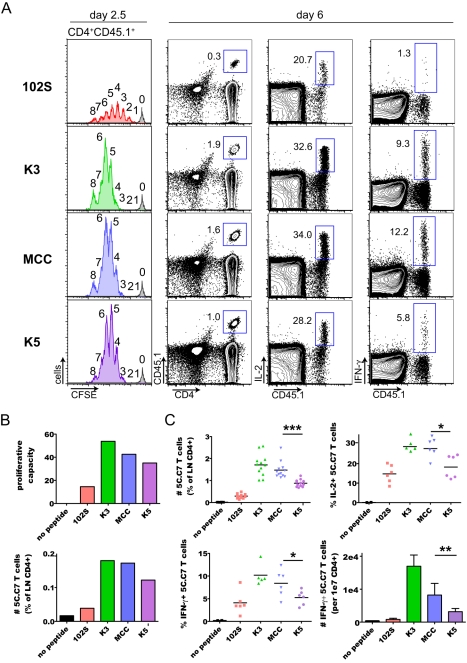
Blunted in vivo responses of 5C.C7 T cells to a high-affinity ligand. Naïve 5C.C7 RAG2−/− CD45.1 T cells were adoptively transferred into normal B10.A recipients and activated by immunization with the indicated peptides and LPS. (A) Day 2.5 CFSE plots represent samples pooled from four or five mice, and number of divisions is indicated at the top of each peak. Frequency of 5C.C7 T cells as a percentage of total CD4^+^ T cells, or frequency of 5C.C7 T cells producing IL-2 or IFN-γ, are shown in samples from day 6 lymph nodes on representative flow plots. (B) Proliferative capacity was calculated from CFSE profiles, and 5C.C7 T cells were quantified as a percentage of total CD4^+^ T cells. Because of the low frequency of 5C.C7 T cells present at day 2.5 it was necessary to combine samples from multiple mice to visualize CFSE profiles and analyze proliferative capacity. Thus, each bar in the graphs represents samples pooled from four or five mice, and the data are representative of three independent experiments. (C) Upper left, number of 5C.C7 T cells (normalized to total CD4^+^ T cells) in day 6 lymph nodes; the data are pooled from four independent experiments. Horizontal lines on graphs represent the mean. ***p<0.0001. Upper right and lower left, number of IL-2^+^ and IFN-γ^+^ cells (as a percentage of 5C.C7 T cells). IL-2, *p = 0.0104. IFN-γ, *p = 0.0463. The lower right panel shows the absolute number of IFN-γ^+^ 5C.C7 T cells per 1×10^7^ lymph node CD4^+^ T cells. **p = 0.0078. Cytokine data are pooled from two independent experiments. Two-tailed *p* values are from unpaired *t* test.

**Figure 5:**
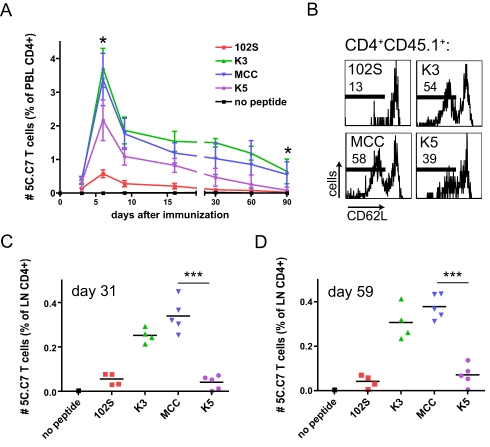
Fewer K5-stimulated 5C.C7 T cells are found at the peak, contraction, and maintenance phases of the response. 5C.C7 T cells were adoptively transferred and activated as in Figure 3, and blood samples were analyzed for CD4^+^ CD45.1^+^ cells at the indicated time points (A). Number of 5C.C7 T cells expressed as a percentage of total CD4^+^ T cells is shown. Error bars show mean ± s.d. Day 6, *p = 0.0247, *n* = 5. Day 90, *p = 0.0257, *n* = 5. Data are representative of two independent time courses. (B) shows CD62L staining of day 59 5C.C7 T cells from lymph nodes. Number of 5C.C7 T cells in lymph nodes is shown at day 31 (C, ***p<0.0001) and day 59 (D, ***p<0.0001). Two-tailed *p* values are from unpaired *t* test. Data in (B), (C), and (D) are representative of two or more independent experiments.

In vivo activation of small numbers of adoptively transferred 5C.C7 T cells by immunization with MCC peptide and LPS reproducibly results in T cell expansion, contraction, and maintenance (Figure 3) [31]. At the peak of the expansion (6–7 d after immunization) the 5C.C7 T cells represented up to 8% of CD4^+^ T cells in peripheral blood, and after contraction were maintained at 1%–2% of blood CD4^+^ T cells (Figure 3B). When we compared in vivo 5C.C7 T cell responses to the various peptide ligands, we saw that K3 and MCC agonist peptides induced greater CFSE dilution, peak expansion, and maintenance of 5C.C7 T cells than the weak agonist 102S peptide (Figures 4 and 5), consistent with their greater in vitro potency. Surprisingly, we found that the in vitro–defined superagonist K5 peptide induced the accumulation of fewer 5C.C7 T cells than MCC at every phase of the immune response (Figures 4 and 5). This correlated with slightly delayed CFSE dilution and decreased proliferative capacity of K5-stimulated cells at early times after immunization (Figure 4A,B).

To examine the possibility that high-avidity interactions with APC make in vivo K5-stimulated 5C.C7 T cells more difficult to extract during homogenization of lymph node tissue prior to flow cytometric analysis [32], accounting for the decreased numbers of 5C.C7 T cells in lymph nodes after K5 immunization, we examined CD45.1 immunofluorescence on cryosections of day 6 lymph nodes from mice immunized with MCC or K5 peptide (Figure S3). Figure S3A shows five representative images each from MCC and K5 immunizations. Using Metamorph software, the number of CD45.1^+^ 5C.C7 T cells was estimated within T cell areas by dividing the total PE fluorescence by the average individual 5C.C7 T cell area (defined by lack of B220 and CD19 staining) in MCC- or K5-stimulated samples (Figure S3B and Materials and Methods). The immunofluorescence data are consistent with the flow cytometry results in Figure 4 and show that there are fewer K5-stimulated 5C.C7 T cells per mm^2^ of T cell area (Figure S3B). K5-stimulated 5C.C7 T cells appear to have, on average, a smaller individual cell area than MCC-stimulated cells (Figure S3C) and less CD45.1 (Figure S3A and D), which was taken into account as part of the quantitation shown in Figure S3B.

Upon ex vivo restimulation of CD4^+^ T cells from day 6 lymph nodes, 5C.C7 T cells activated with any of the four peptides produced interleukin-2 (IL-2) and interferon-γ (IFN-γ) (Figure 4A,C). For 102S, K3, and MCC peptides, the number of cytokine-producing 5C.C7 T cells correlates with in vitro ligand potency; however, fewer K5-stimulated 5C.C7s produced IL-2 and IFN-γ when compared to MCC-stimulated cells (Figure 4A,C), showing that the effector function of K5-stimulated cells is diminished in addition to proliferation. Thus, fewer 5C.C7 T cells accumulate in response to immunization with K5 peptide as compared to MCC peptide, and fewer of those secrete IFN-γ in an ex vivo restimulation assay. The number of IFN-γ^+^ 5C.C7 T cells induced in response to immunization with the various ligands is shown in the lower right panel of Figure 4C. The number of IFN-γ secreting 5C.C7 T cells is 60% reduced in K5-immunized mice, when compared to MCC immunization. Such a reduction is likely to affect the inflammatory milieu, given that IFN-γ is a key mediator of many aspects of immunity.

No significant differences were found in levels of canonical activation markers such as CD25, CD62L, and CD44 during the initial expansion of 5C.C7 T cells in response to the various peptides (Figure S4A,C). However, a lower percentage of resting memory 5C.C7 T cells initially stimulated with 102S and K5 peptides have a CD62L^lo^ phenotype (Figure 5B), suggesting that, in addition to lower numbers of total memory cells in blood (Figure 5A) and lymph nodes (Figure 5C,D), these peptides may induce a lower fraction of effector memory cells (T_EM_) [33]–[35] than MCC (although other possibilities exist, see Discussion). When the resting memory cells generated with the various peptides were restimulated with MCC peptide, the results were similar to those in the primary stimulation (Figure S5A,B), which likely reflects precursor frequency [36]. Similar percentages of memory cells generated with K5 and MCC peptides are able to produce IFN-γ upon ex vivo restimulation (Figure S5C), suggesting that the memory cells generated with K5 peptide are as functional on a per-cell basis as those generated with MCC, even though they are maintained in lymph nodes at a much lower frequency (Figure 5C,D). Thus, ligand potency affects the early in vivo division, accumulation, and cytokine production of a monoclonal population of CD4^+^ T cells. In contrast to what is predicted from biochemical data, the strong peptide ligand K5 induces a blunted in vivo 5C.C7 response that is of lower magnitude and function than the wild-type ligand MCC.

### K5 pMHC Complexes Are Not Limiting In Vivo

Since endogenous T cells have been shown to compete with adoptively transferred T cells for cognate pMHC-bearing APCs [37], we examined responses to the various peptides by MCC/I-E^k^ tetramer staining of lymph node cells from B10.A mice (containing no adoptively transferred 5C.C7 T cells). Seven days after immunization, we found low percentages of endogenous responder T cells to all four peptides (<0.1% of total CD4^+^ T cells; Figure S6). A more competitive endogenous response is therefore unlikely to be the explanation for the decreased responses of 5C.C7 T cells to K5 peptide in vivo. Also, titration of peptides in vivo showed that there was no peptide dose at which K5 peptide induced more proliferation than MCC (unpublished data).

We addressed potential differential in vivo antigen persistence by injecting MCC or K5 peptide at various times prior to transfer of CFSE-labeled 5C.C7 T cells (Figure S7). In this experiment, the extent of division of the transferred T cells serves as a readout of amount of antigen present. The data show that MCC and K5 peptides persist equally and for a few weeks in vivo. We then considered the possibility that the blunted responses were occurring because the amount of K5 pMHC complexes was limiting in vivo. In order to directly examine the stimulatory function of in vivo–pulsed APCs, we used irradiated splenic DCs isolated from mice immunized with the different peptides to activate naïve 5C.C7 T cells in vitro, without the addition of exogenous peptide (Figure 2B,C). The rank order of T cell activation potency of the peptides in this assay was the same as in the in vitro proliferation assay shown in Figure 2A, which demonstrates that presentation of K5 peptide is not diminished in vivo. Transfer of 5C.C7 T cells does not affect the results of the experiment (Figure 2B). These results also show that K5 pMHC complexes assembled in vivo are high avidity 5C.C7 T cell ligands and that the blunted in vivo responses to K5 peptide are not due to attenuation of APC maturation or function, since the APCs from mice immunized with K5 peptide are equally capable of presenting antigen (Figure 2D). Thus, the diminished responses to K5 peptide in vivo are not recapitulated in vitro when in vivo–pulsed APCs are used, which suggests that T cell activation is attenuated at some point after initial high-avidity ligand recognition in vivo. Indeed, at early time points after activation, 5C.C7 T cells stimulated in vivo have higher levels of CD5 (Figure S4B), which is consistent with their experience of a stronger TCR signal in vivo [38]–[41].

We also did not find evidence that the blunted responses to K5 peptide are due to preferential suppression of the 5C.C7 effector T cells by regulatory T cells. When CD4^+^CD25^+^Foxp3^+^ cells were depleted by injection of anti-CD25 antibody (Figure S8), numbers of MCC- and K5-stimulated 5C.C7 T cells in lymph nodes at the peak of the response are indistinguishable from controls (Figure S5E). The levels of regulatory T cell depletion observed after injection of anti-CD25 antibody (one third of initial levels in lymph nodes; Figure S8D) are sufficient to significantly enhance tumor-reactive T cell responses in mice [42],[43]. Subcutaneous immunization with K5 peptide in the presence of LPS does not result in expression of Foxp3 by 5C.C7 T cells (Figure S9). This is in contrast to what we observe during intravenous injection 5C.C7 T cell ligands in the absence of LPS [44]. In summary, the lower capacity for expansion, cytokine production, maintenance, and effector memory differentiation displayed by the K5-stimulated cells is not explained by competitive K5-reactive endogenous T cell responses, antigen dosage or persistence effects, lower in vivo stability of the K5 peptide (or pMHC complexes), or enhanced suppression of high-avidity T cell responses by regulatory T cells.

### In Vivo, K5-Stimulated 5C.C7 T Cells Have Decreased Levels of Phosphorylated Signaling Intermediates Compared to Those Stimulated with MCC

**Figure 6:**
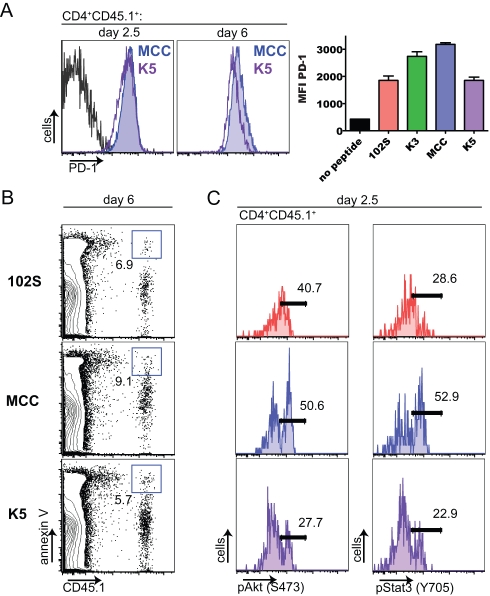
In vivo activation status of 5C.C7 T cells stimulated with the high-affinity ligand K5 peptide. Naïve 5C.C7 RAG2−/− CD45.1 T cells were adoptively transferred and activated by immunization with the indicated peptides and LPS. (A) Lymph node CD4^+^ T cells were stained for PD-1 at day 2.5 and day 6 (CD4^+^CD45.1^+^ gated). The day 2.5 histograms represent samples pooled from four or five mice. The black histogram corresponds to cells stimulated with LPS alone. Graphed PD-1 MFIs are from day 6, error bars show mean ± s.d., *n* = 3 mice. (B) Annexin V staining on CD4^+^ T cells from day 6 lymph nodes. (C) Day 2.5 lymph node samples were fixed immediately after harvest, methanol permeabilized, and stained with antibodies to phosphorylated Akt (S473) and phosphorylated Stat3 (Y705). Histograms are gated on CD4^+^CD45.1^+^ cells and represent samples pooled from four or five mice. Percent of 5C.C7 T cells positive for annexin V, pAkt, or pStat is shown on the plots and histograms. The data are representative of three independent experiments.

When we further investigated the phenotype of K5-stimulated 5C.C7 T cells, we found no evidence of clonal exhaustion as defined by increased expression of PD-1 (Figure 6A) or increased amounts of apoptosis (Figure 6B) compared to MCC-stimulated cells. In addition, the possibility that K5-stimulated 5C.C7 T cells divide more rapidly than MCC-stimulated cells and are subsequently deleted [45] is not consistent with the delay in CFSE dilution exhibited by K5-stimulated cells at early time points (Figure 4A). Taken together, these results indicate that K5-stimulated 5C.C7 T cells are not likely to be abortively over-activated in vivo.

Our data suggest that T cell responses to high-avidity ligands are attenuated in vivo. CTLA-4 is a well-characterized negative regulator of T cell activation that has been suggested to preferentially restrict T cell responses to strong TCR signals [46]. However, we found no evidence that inhibition by CTLA-4 is involved in the blunted T cell responses we observe to K5 peptide in vivo (E.C. and J.P.A., manuscript in preparation).

To gain insight into the molecular mechanisms of attenuation that occur in response to stimulation with K5 peptide, we fixed lymph nodes immediately after sacrifice (60 h after immunization) and analyzed the in vivo activated 5C.C7 T cells for levels of phosphatases and phosphorylated signaling intermediates (Figure 6C and Figures S10 and S11). The SH2-domain containing tyrosine phosphatase (SHP-1) is thought to participate in a negative feedback loop that functions in TCR ligand discrimination [47], but we did not observe significant differences in levels of either SHP-1 or phosphorylated ERK between MCC- and K5-stimulated cells at the time points examined (Figure S10). This could be because regulation of TCR ligand discrimination by SHP-1 levels is restricted to the boundary between low-affinity agonists and non-agonists/antagonists, as has been suggested [48]. We also did not detect increased levels of the lipid phosphatase PTEN, which negatively regulates the PI3 kinase pathway [49], in K5-stimulated 5C.C7 T cells (Figure S10).

Strikingly, when the in vivo–activated 5C.C7 T cells were analyzed for levels of S473-phosphorylated Akt (pAkt), which reflects optimally active Akt kinase [50], and phosphorylated Stat3 (pStat3), which is induced in response to multiple cytokines [51] (Figure 6C, Figure S11), fewer K5-stimulated 5C.C7 T cells were positive for pAkt and pStat3, compared to MCC-stimulated cells (Figure 6C). The decreased amounts of signaling intermediates in two important pathways in the K5-stimulated 5C.C7 T cells suggest that their activation state is negatively regulated on an intracellular level in response to high-avidity TCR stimulation in vivo. Upon in vitro stimulation, more K5- than MCC-stimulated 5C.C7 T cells are positive for pAkt and pStat3 (Figure S12), consistent with the greater in vitro potency of K5 peptide (Figure 2). These results reveal a clear difference between the in vivo and in vitro regulation of the T cell response to a high-avidity ligand.

## Discussion

In contrast to the assumption that more potent T cell responses result from higher-avidity TCR recognition, here we report diminished T cell responses to a strong TCR ligand in vivo. We studied the in vivo activation of 5C.C7 T cells by immunization with LPS and a panel of peptides with a range of in vitro T cell activation potency, which results in fully functional effector and memory responses. Interestingly, we observed optimal T cell responses to the peptide ligands with intermediate TCR/pMHC half-lives. Contrary to what is predicted from in vitro data, the K5 ligand, which has the longest TCR/pMHC half-life, induces less in vivo division, accumulation, cytokine production of 5C.C7 T cells during the effector phase of the response, as well as persistence of fewer 5C.C7 T cells during the memory phase.

Given that we observe fewer CD62L low memory cells in lymph nodes after immunization with K5 peptide, we cannot rule out that K5 favors T_EM_ development and greater homing to tissues [34], thus accounting for the fewer numbers of memory 5C.C7 T cells in lymph nodes prior to exit (Figure 5B). The data in Figure 5B would also be consistent with accelerated T_CM_ development, or even impaired T_EM_ development if the CD62L low population present in the MCC response is considered to be lymph node T_EM_ cells (Figure 5B). Increased T_EM_ development and tissue homing induced by K5 peptide could potentially account for the decreased numbers of K5-stimulated T cells at later time points, but it is unlikely that it could explain the delayed CFSE dilution and decreased accumulation in lymph nodes at the peak of the primary response, observed at day 2.5 and day 6, respectively (Figure 4 and Figure S4C), especially given the lack of evidence for increased egress of K5-stimulated cells into the blood at these earlier time points (Figure 5).

We found that the blunted response to K5 peptide is not due to a lack of in vivo availability of peptide/MHC complexes, dose effects, or competition by endogenous responses. In addition, the 5C.C7 T cells stimulated with K5 peptide in vivo do not show signs of apoptosis or clonal exhaustion, making it unlikely that they are abortively over-activated. Finally, the K5-stimulated 5C.C7 T cells are not preferentially inhibited by regulatory T cells or by the T cell inhibitory receptor CTLA-4. Interestingly, we observed attenuation at the level of intracellular signaling intermediates associated with Akt kinase activation and cytokine signaling, which we measured directly ex vivo using antibodies to pAkt and pStat3. Fewer K5-stimulated 5C.C7 T cells were positive for pAkt, indicating decreased activity of the mammalian target of rapamycin (mTOR)-containing complex mTORC2 in these cells [50]. We also observed fewer pStat3-positive K5-stimulated 5C.C7 T cells, which may indicate that the cells are less responsive to cytokines which activate the Stat3 pathway [51].

In a recent study of in vivo CD8^+^ T cell activation, in vitro ligand potency correlated with the magnitude of the in vivo T cell response, i.e. the highest-affinity ligand (which was the natural OT-1 ligand SIINFEKL) induced the most robust response [25]. The reason we observed attenuation at the high end of the ligand potency spectrum may be because we were able to examine T cell responses to a superagonist peptide, which possibly exceeds a ligand potency threshold at which T cell attenuation is observed. Attempts to find other strong 5C.C7 TCR ligands, which would facilitate examination of this hypothesis, have been unsuccessful thus far. It is also possible that the attenuation we observed in vivo in response to high-avidity TCR stimulation is unique to CD4^+^ T cell responses.

The effect of adjuvant upon the avidity of TCRs in the polyclonal CD4^+^ T cell response to pigeon cytochrome c (PCC) protein was recently examined by Malherbe et al. [52]. These authors found that immunization in the presence of non-depot forming adjuvants containing toll-like receptor 4 or 9 agonist resulted in accumulation of high-avidity PCC-reactive T cells at the peak of the response, based on their previous characterization of the avidity of TCRs bearing specific CDR3 sequences [53], as well as levels of pMHC II tetramer binding. The presence of high-avidity CD4+ T cell clones in the Malherbe et al. study is not inconsistent with attenuation of the monoclonal CD4^+^ T cell population that we tracked in response to strong TCR ligand. It is hard to compare the avidities of the responding TCRs since both the TCRs and the peptide ligands in question are different. Therefore, it is possible that the responses to the strongest ligand in our study exceeds an avidity threshold beyond which T cell attenuation occurs, and that this threshold is not reached by any of the TCRs in the Malherbe et al. study. It is also possible that the naïve repertoire in the Malherbe et al. experiments contains T cells with even higher avidity for PCC antigen than the clones favored by immunization with the TLR agonists. These clones may then be attenuated in response to high-avidity TCR engagement, such as we observed in our study. This could result in the underrepresentation of such clones at the peak of the primary response, and thus they may not be present during the CDR3 sequence analysis performed by Malherbe et al. [52].

At the low potency end of the ligand spectrum, our results were in agreement with the CD8^+^ T cell study mentioned above [25]; the lowest affinity ligand we examined (102S) was able to induce a functional response. Interestingly, we saw that the lower-magnitude T cell response induced by 102S correlates with low levels of pAkt and pStat3 before the expansion phase; however, it is possible that the accumulation of these phosphorylated intermediates occurs at a later time in response to the weak agonist peptide [54]. The observation of sustained signaling intermediates such as pAkt in the in vivo T cell response to antigen is not necessarily predicted by in vitro studies [55] and may point to the importance of the cumulative activity of signaling pathways in defining the magnitude of in vivo T cell responses. Thorough examination of the timing of engagement and sustained activity of signaling pathways downstream of in vivo T cell activation is likely to lend further insight into such questions, as well as the biochemistry of in vivo T cell activation in general.

We propose that negative regulation of the extent of T cell activation occurs in response to high-avidity in vivo TCR stimulation, potentially in the context of the long-lived T cell-DC contacts that are a hallmark of in vivo antigen recognition [56], and that this is characterized by but not necessarily limited to a decrease in the phosphorylated signaling intermediates we measured in this study. Although we did not find increased levels of the inhibitory phosphatases SHP-1 and PTEN in the K5-stimulated cells, cell-intrinsic attenuation of activation could be certainly mediated by other intracellular negative regulators of TCR signaling [57] and/or increased internalization and degradation of cell surface receptors [58] in response to strong sustained in vivo TCR engagement. We did not observe increased TCR downregulation by K5-stimulated cells (unpublished data). The strong in vivo TCR signal could also influence (directly or indirectly) induction of or responsiveness to cytokines, resulting in attenuated T cell responses. Although we did not find a role for regulatory T cells in the blunted response to K5 peptide in experiments where CD4^+^CD25^+^Foxp3^+^ cells were depleted by injection of anti-CD25 antibody (Figure S8), nor did we observe Foxp3+ induction by 5C.C7 T cells in response to subcutaneous immunization with K5 (Figure S9), other cell-extrinsic mechanisms of attenuation cannot be excluded.

Attenuation of in vivo T cell responses to high-avidity pMHC ligands may function to prevent detrimental inflammatory effects that could occur in response to high-avidity TCR recognition of either self or foreign peptides. Our results are contrary to the common assumption that higher-avidity TCR recognition promotes a more potent T cell response and point to an upper limit of T cell ligand potency in vivo. Interestingly, a study of a panel of peptide ligands with variable affinity for a tumor-reactive TCR showed that elicitation of the most potent tumor-reactive CD8^+^ T cells in vivo and the best tumor-free survival occurred after vaccination with peptide ligands that displayed intermediate affinity and in vitro T cell activation potency [11]. Although it is unclear if the tumor-reactive T cells in vivo bear the same TCR as the clone on which ligand affinity was characterized, the results point out an important discrepancy between the in vitro and in vivo efficacy of TCR ligands. This discrepancy, along with the attenuation we observed in vivo while tracking the responses of monoclonal CD4^+^ T cells to a strong ligand, suggests that vaccination strategies that aim to elicit maximum-avidity TCR recognition with engineered peptide ligands may warrant reconsideration [11].

## Materials and Methods

### Ethics Statement

All mice were maintained in microisolator cages and treated in accordance with NIH and the American Association of Laboratory Animal Care regulations. Experiments in this study were approved by the Memorial Sloan-Kettering Cancer Center Institutional Animal Care and Use Committee.

### Purification of Soluble 5C.C7 TCR and SPR

Coding regions for the 5C.C7 TCR α and β chains were cloned by PCR into a modified version of the transfer vector pACGP67A (Pharmingen), which placed the 5C.C7 α and β in frame with c-jun (α) and c-Fos (β) heterodimerization motifs. Both chains were linked at the C-terminus to a 6XHis tag through a 3C-protease-cleavable site. TCR was expressed in insect cells as previously described [23], isolated from the supernatant by Ni-NTA purification, cleaved with 3C protease, and further purified by ion-exchange and size exclusion chromatography. Protein concentration was determined using an extinction coefficient (280 nm) of 1.3 ml⋅mg^1^⋅cm^1^. All SPR data were collected at 25°C using a BIAcore 3000™ instrument (BIAcore Inc.). Purified biotinylated pMHC complexes were immobilized on a streptavidin chip (BIAcore Inc.) to a level of 250–500 RU. Free biotin-binding sites were blocked with 10 mM biotin. Binding of 5C.C7 TCR was measured in PBS, pH 7.8 with 0.005% Surfactant P20 (BIAcore Inc.) by injecting a range of concentrations (0.9–14.3 µM) at a flow-rate of 30 µl/min. All data were background subtracted using a biotin-blocked streptavidin surface as a reference surface. Data were fitted to a Langmuir binding model using global fitting (Biaevaluation, BIAcore Inc.) to calculate k_a_ and k_d_ constants. To prevent aggregation artifacts, all proteins were purified no more than 12 h before analysis without subsequent concentration steps.

### Mice

5C.C7 TCR transgenic RAG2−/−- mice were purchased from Taconic Farms and bred to B10.A CD45.1 (provided by W. Paul via the NIAID contract facility at Taconic Farms) to generate 5C.C7 RAG2−/− CD45.1 mice, which were used as donor cells in adoptive transfer experiments. Male B10.A recipient mice were purchased from Taconic and were used to initiate experiments at 6–9 wk of age.

### Tetramers, Tetramer Dissociation, and Apparent K_D_ Analysis

Tetramer production, dissociation assays, and data analysis were done as described [27]. Briefly, PE-labeled I-E^k^ tetramers with MCC and variant peptides were produced. Naïve 5C.C7 T cells were incubated with tetramers (50 µg/mL) for 2 h at RT in the presence of fresh 0.2% NaN_3_, equilibrated to 37°C or 25°C, and measurement of dissociation was initiated with the addition of anti-I-E^k^ antibody. Aliquots were removed in 10–20 s intervals and fixed immediately in 1% PFA. Levels of tetramer staining were assessed by flow cytometry. Apparent K_D_ analysis was done as previously described [27]. Briefly, varying concentrations of tetramers were used to stain the 5CC7 T cells as described above, and the median intensity of tetramer staining was subjected to Scatchard analysis. Since tetramers were always in excess, free tetramer concentration is equal to staining concentration.

### Cell Culture and In Vitro T Cell Activation

Cells were cultured in a 37°C humidified chamber with 5% C0_2_ in complete RPMI1640 (supplemented with 10% FCS, 2 µM glutamine, 100 U/mL penicillin and streptomycin, 2 µM 2-mercaptoethanol). Single-cell suspensions were prepared from lymph nodes harvested from 5C.C7 RAG2-deficient mice (routinely >90% Vα11+Vβ3+ by flow cytometry) and stimulated in triplicate in 96-well round-bottom plates with irradiated (2,000 rad) B10.A splenocytes and the indicated peptides for 60 h. Proliferation was monitored by the addition of ^3^H-methyl-thymidine (1 µCi/well). Cells were harvested onto glass-fiber filters using a Tomtec harvester, and filters were counted using a MicroBeta scintillation counter (Perkin-Elmer).

### Peptides and LPS

Peptides were synthesized and HPLC-purified (≥95%) by Biosynthesis, Inc. (Lewisville, TX). Purified LPS was from Invivogen.

### Flow Cytometry and Antibodies

Flow cytometry was done on a BD LSRII and data were analyzed with Flowjo software (Treestar). Antibodies to surface markers were from BD Pharmingen, eBioscience, or BioLegend. Antibodies to phospho-Akt, phospho-Stat, phospho-Erk, and PTEN were Alexa 647 conjugates from Cell Signaling Technology. SHP-1 antibody was from Santa Cruz Biotechnology.

### In Vivo T Cell Activation

1–5×10^4^ 5C.C7 RAG2−/− CD45.1 T cells were transferred into B10.A recipients by tail vein injection, and the mice were immunized the next day with 20 µg peptide and 10 µg LPS at two sites on either side of the base of the tail. In some experiments the cells were CFSE-labeled prior to transfer. Proliferative capacity was calculated from CFSE profiles as described [59]. Total lymph nodes were pooled for all experiments. Cytokine production was assessed by ex vivo restimulation for 4–5 h with B10.A DCs and peptide and intracellular cytokine staining using BD Biosciences reagents.

### Immunofluorescence

5C.C7 RAG2−/− CD45.1 T cells were activated in vivo as described above. Six days after immunization with MCC or K5 peptide (5 mice per peptide), mice were sacrificed and the lymph nodes were embedded directly into OCT and frozen using dry ice and methanol. After temporary storage at −80°C, 10 µm sections were cut through the entire block (containing 50 lymph nodes) using a Leica Cryostat and stained with anti-CD45.1-biotin/streptavidin-PE to mark 5C.C7 T cells and anti-B220-FITC and anti-CD19-FITC to mark B cell areas. The stained sections (each containing 10 or more lymph nodes) were scanned with a Mirax slide scanner (epifluorescence). The number of 5C.C7 T cells was quantified from the T cell areas of 20 independent lymph nodes using Metamorph software. Total fluorescence was divided by the average individual T cell area to estimate the number of 5C.C7 T cells per mm^2^ of T cell area.

### CD4+ Purification from Lymph Node Suspensions

To enrich for 5C.C7 T cells originating from low precursor frequency before flow cytometry, CD4+ T cells were purified from lymph node suspensions by Dynal negative selection (Invitrogen).

### Analysis of Phospho-Intermediates

Lymph node suspensions were fixed immediately after harvest and then negatively selected for CD4+ T cells. After staining with antibodies to CD4 and CD45.1, cells were permeabilized with methanol and stained with phospho-antibodies.

### In Vivo–Pulsed APCs

CD11c^+^ cells were purified by positive selection (Miltenyi) from spleens of mice adoptively transferred and immunized as described, irradiated, and used to stimulate naïve 5C.C7 RAG2−/− T cells in vitro. Proliferation was assessed by ^3^H-thymidine incorporation as described.

### Statistical Analysis

Data were analyzed for significance using unpaired Student's *t* test analysis with Prism software.

## Supporting Information

Figure S1 **Correlation of 5C.C7 TCR/peptide-I-E^k^ half-life derived by tetramer dissociation and SPR.** Plot of the half-life of interaction of purified 5C.C7 TCR with I-E^k^ complexed with K3, MCC, and K5 peptides derived by SPR (Figure 1A–C) versus the half-life derived by tetramer dissociation (Figure 1D).
(0.45 MB EPS)

Figure S2 **K5/I-E^k^ tetramers interact more strongly with 5C.C7 T cells by equilibrium tetramer staining and apparent K_D_ analysis.** (A) Naive 5C.C7 T cells were stained for 2 h at 25°C with the indicated I-E^k^ tetramers (50 mg/mL). *p = 0.0190. Data are representative of three independent experiments and error bars show mean ± s.d. (B) Scatchard plot of tetramer binding at varying concentrations of tetramer, expressed in nM. Apparent K_D_ analysis was done as described in Materials and Methods and [27]. Data are representative of two independent experiments, K_D_ values from which are shown in (C). *p = 0.0364. Two-tailed *p* value is from unpaired *t* test.
(0.82 MB EPS)

Figure S3 **Immunofluorescence of 5C.C7 T cells in lymph nodes 6 d after immunization with MCC or K5 peptide.** B10.A mice adoptively transferred with CD45.1^+^ 5C.C7 RAG2−/− T cells were immunized with LPS and MCC or K5 peptide as described in Materials and Methods. Six days later, lymph nodes were embedded in OCT, frozen, sectioned, stained, and imaged as described in Materials and Methods. (A) Five representative images from the lymph nodes of MCC- and K5-immunized mice are shown. B cells are green and 5C.C7 T cells are red. The number of 5C.C7 T cells in T cell areas was quantitated as described in Materials and Methods. The data comprise analysis of the T cell areas from 20 independent lymph nodes per peptide, randomly sampled from 50 lymph nodes and five independent mice per peptide. (C) Area measurements of individual 5C.C7 T cells stimulated with MCC or K5 peptide. (D) The dim αCD45.1 immunofluorescence staining apparent in the lymph nodes of K5-stimulated mice is consistent with that seen during flow cytometric analysis. The graph shows the MFI (by flow cytometry) of CD45.1 from day 6 lymph node samples and represents 12 independent mice per peptide. Two-tailed *p* values are from unpaired *t* test.
(7.04 MB TIF)

Figure S4 **Phenotype of in vivo stimulated 5C.C7 T cells.** (A) B10.A mice with adoptively transferred CD45.1^+^ 5C.C7 RAG2−/− T cells were immunized with indicated peptides and LPS, and CD4^+^ T cells from lymph node were analyzed 2.5 d after immunization. Each plot represents samples pooled from three to five mice. Percent of CD25^+^ or CD62Llo (of CD4^+^CD45.1^+^ cells) is shown on plots. (B) CD5 levels on 5C.C7 T cells 2.5 d after immunization. Each histogram and bar represent samples pooled from three to five mice. (C) Lymph node samples analyzed at day 6. Error bars show mean ± s.d., *n* = 3 mice.
(2.88 MB EPS)

Figure S5 **Reactivation of resting lymph node 5C.C7 T cells.** B10.A mice with adoptively transferred CD45.1^+^ 5C.C7 RAG2−/− T cells were immunized with indicated peptides and LPS. (A) PBLs 45 d later immunization. *p = 0.0332, *n* = 4. (B) The mice in (A) were all reimmunized with MCC peptide and LPS, and lymph node CD4^+^s were analyzed 4 d later. **p = 0.0061, *n* = 4. (C) Lymph node CD4^+^s from the mice in (B) were restimulated ex vivo with DCs and MCC peptide and stained for intracellular IFN-γ. Two-tailed *p* values are from unpaired *t* test.
(0.59 MB EPS)

Figure S6 **Endogenous responses to MCC and related variant peptides.** B10.A mice were immunized with the indicated peptides and LPS, and lymph node cells were stained with PE-MCC/I-E^k^ tetramer 7 d later. (A) Plots are gated on CD4^+^CD44^hi^B220-propidium iodide. Percent of tetramer^+^ cells (of CD4^+^ T cells) is shown on plots and graphed in (B). Horizontal lines on the graph represent the mean. (C) Mean fluorescence intensity of tetramer in gated populations. Error bars show mean — s.d., *n* = 3 mice. The data are representative of two independent experiments.
(1.81 MB EPS)

Figure S7 **MCC and K5 peptides persist equally in vivo.** B10.A mice were immunized with the indicated peptides at 4, 2, 1, and 0 wk before adoptive transfer of CFSE-labeled 5C.C7 T cells. 5C.C7 T cell division was analyzed at 48 h after transfer as a measure of peptide antigen persistence over time. All histograms are gated on CD4^+^CD45.1^+^ 5C.C7 T cells. Data shown are representative of two mice per condition and two experiments.
(0.99 MB EPS)

Figure S8 **No evidence of a role for regulatory T cells in blunted responses to K5 peptide in vivo.** B10.A mice were injected intraperitoneally with 0.5 mg αCD25 antibody (clone PC61) to deplete regulatory T cells, or 0.5 mg rat IgG as a control. (A,B) Three days later, mice were bled and PBLs were analyzed for CD4^+^CD25^+^Foxp3^+^ cells to confirm depletion, and adoptively transferred with 5C.C7 T cells. (C,D) Analysis of CD4^+^Foxp3^+^ cells in lymph node samples 10 d after depletion shows decreased amounts of regulatory T cells persist throughout the course of the experiment. (E) Seven days after immunization with either MCC or K5 peptide (11 d after injection of PC61), CD45.1^+^ 5C.C7 T cells were quantified in lymph node samples. Data are representative of three independent experiments.
(1.84 MB TIF)

Figure S9 **Subcutaneous immunization with peptide and LPS does not induce Foxp3 expression in 5C.C7 T cells.** B10.A mice containing adoptively transferred CD45.1^+^ 5C.C7 T cells were immunized subcutaneously with LPS+the indicated peptide. Six days after immunization, Foxp3 expression was assessed in lymph node samples by flow cytometry. Each histogram represents an individual mouse, and the experiment shown is representative of two independent experiments. As a control, the bottom histogram shows the induction of Foxp3 that occurs in 5C.C7 T cells in response to low doses of i.v. MCC peptide.
(1.59 MB EPS)

Figure S10 **No significant differences in ppErk, SHP-1, and PTEN levels between MCC- and K5-stimulated 5C.C7 T cells.** B10.A mice containing adoptively transferred 5C.C7 T cells were immunized with LPS+MCC or K5 peptide. 2.5 d after immunization, lymph node samples were fixed immediately after harvest, methanol permeabilized, and stained with fluorophore-conjugated antibodies to ppErk, SHP-1, or PTEN (C). Histograms are gated on CD4^+^CD45.1^+^ cells (A). The graph in (C) shows that fewer K5 stimulated 5C.C7 T cells are present at the 2.5 d time point. The black histograms in (C) show the levels of the ppErk, SHP-1, and PTEN in endogenous CD4^+^ T cells.
(0.86 MB EPS)

Figure S11 **Specificity controls for phosphoantibodies.** B10.A mice containing adoptively transferred 5C.C7 T cells were immunized with LPS alone or LPS+MCC peptide. 2.5 d after immunization, lymph node samples were fixed immediately after harvest, methanol permeabilized, and stained with the indicated phospho-antibodies. In the right panels, the antibodies were incubated with a peptide corresponding to the reactive phospho-epitope (according to the manufacturer's protocol) prior to staining of lymph node samples. (A) pAkt (S473). (B) pStat3 (Y705).
(0.92 MB EPS)

Figure S12 **pAkt and pStat staining of in vitro–stimulated 5C.C7 T cells.** Lymph node cells from 5C.C7 RAG2−/− mice were stimulated with irradiated CD11c+ splenic DCs and the indicated peptide for 60 h. Cells were fixed, methanol permeabilized, and stained with antibodies to pAkt (S473) (A) or pStat3 (Y705) (B). Histograms are gated on CD4+ cells and percentages of phospho-Akt or phospho-Stat3 are shown.
(0.82 MB EPS)

## Abbreviations

APCs: antigen presenting cells
IFN-γ: interferon-γ
IL-2: interleukin-2
LPS: lipopolysaccharide
MCC: moth cytochrome c
mTOR: mammalian target of rapamycin
pAkt: phosphorylated Akt
PCC: pigeon cytochrome c
pMHC: major histocompatibility complex
RU: resonance unit
SPR: surface plasmon resonance
