# Supplementary figures and images for: Attenuated T Cell Responses to a High-Potency Ligand In Vivo
Source: PLoS Biol. 2010 Sep 14;8(9):e1000481. doi: 10.1371/journal.pbio.1000481 (PMC2939023; doi:10.1371/journal.pbio.1000481)

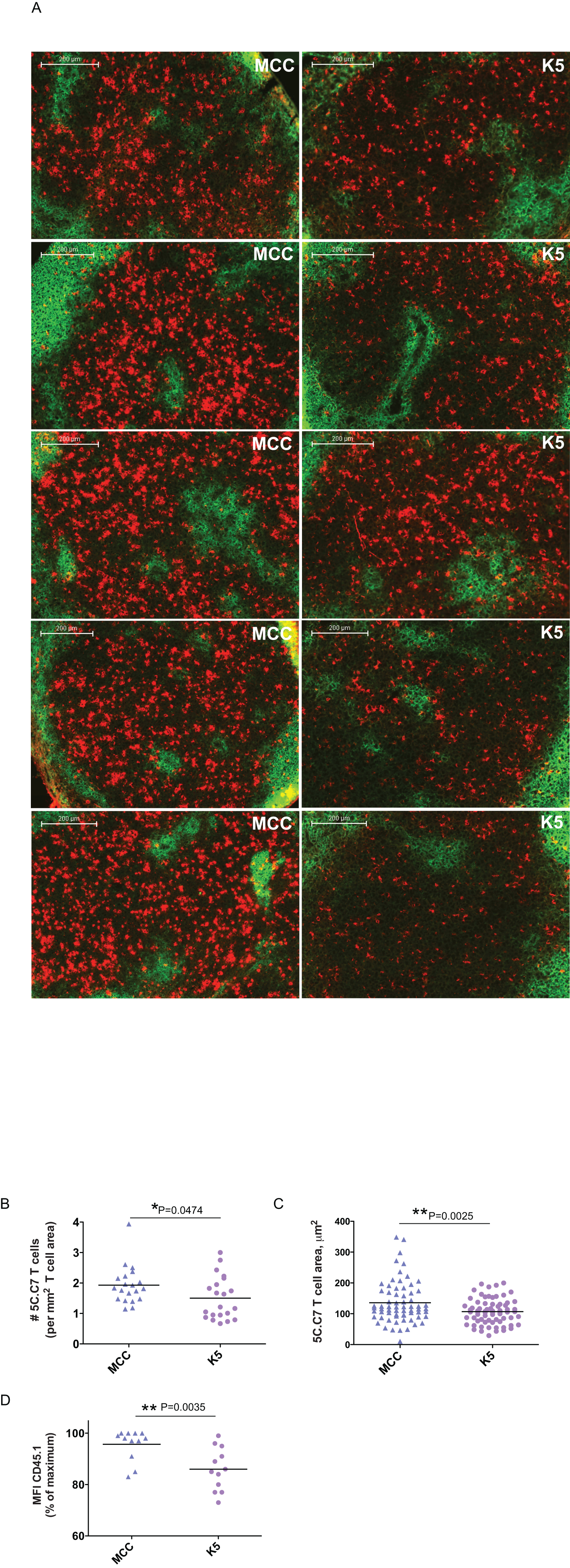

Supplement: Figure S3 — Immunofluorescence of 5C.C7 T cells in lymph nodes 6 d after immunization with MCC or K5 peptide. B10.A mice adoptively transferred with CD45.1+ 5C.C7 RAG2−/− T cells were immunized with LPS and MCC or K5 peptide as described in Materials and Methods. Six days later, lymph nodes were embedded in OCT, frozen, sectioned, stained, and imaged as described in Materials and Methods. (A) Five representative images from the lymph nodes of MCC- and K5-immunized mice are shown. B cells are green and 5C.C7 T cells are red. The number of 5C.C7 T cells in T cell areas was quantitated as described in Materials and Methods. The data comprise analysis of the T cell areas from 20 independent lymph nodes per peptide, randomly sampled from 50 lymph nodes and five independent mice per peptide. (C) Area measurements of individual 5C.C7 T cells stimulated with MCC or K5 peptide. (D) The dim αCD45.1 immunofluorescence staining apparent in the lymph nodes of K5-stimulated mice is consistent with that seen during flow cytometric analysis. The graph shows the MFI (by flow cytometry) of CD45.1 from day 6 lymph node samples and represents 12 independent mice per peptide. Two-tailed p values are from unpaired t test. (7.04 MB TIF) [file pbio.1000481.s003.tif]
